# Metabolomic profiles of plasma and uterine luminal fluids from healthy and repeat breeder Holstein cows

**DOI:** 10.1186/s12917-021-02755-7

**Published:** 2021-01-28

**Authors:** Natsumi Funeshima, Ryotaro Miura, Taiga Katoh, Hikari Yaginuma, Takeshi Kitou, Itaru Yoshimura, Kunitoshi Konda, Seizo Hamano, Koumei Shirasuna

**Affiliations:** 1 Department of Animal Science, Tokyo University of Agriculture, Atsugi, Kanagawa 243-0034 Japan; 2 Department of Veterinary Medicine, Nippon Veterinary and Life Science University, Musashino, Tokyo 180-8602 Japan; 3 Animal Bio-Technology Center, Livestock Improvement Association of Japan Inc., Shinagawa, Tokyo 135-0041 Japan; 4 Fuji Animal Research Farm, Nippon Veterinary and Life Science University, Kawaguchiko, Yamanashi 401-3338 Japan; 5 Kanagawa Prefectural Livestock Industry Technology Center, Ebina, Kanagawa 243-0417 Japan; 6 Maebashi Institute of Animal Science, Livestock Improvement Association of Japan Inc., Maebashi, Gunma 371-0121 Japan

**Keywords:** Bile acids, Interferon tau, Indoleamine 2,3-dioxygenase, Repeat breeding

## Abstract

**Background:**

Repeat breeding is a critical reproductive disorder in cattle. The problem of repeat breeder cattle remains largely unmanageable due to a lack of informative biomarkers. Here, we utilized metabolomic profiling in an attempt to identify metabolites in the blood plasma and uterine luminal fluids. We collected blood and uterine fluid from repeat breeder and healthy cows on day 7 of the estrous cycle.

**Results:**

Metabolomic analysis identified 17 plasma metabolites detected at concentrations that distinguished between the two groups, including decreased various bile acids among the repeat breeders. However, no metabolites that varied significantly were detected in the uterine luminal fluids between two groups. Among the plasma samples, kynurenine was identified as undergoing the most significant variation. Kynurenine is a metabolite produced from tryptophan via the actions of indoleamine 2,3-dioxygenase (IDO). As IDO is key for maternal immune tolerance and induced in response to interferon tau (IFNT, ruminant maternal recognition of pregnancy factor), we examined the responsiveness to IFNT on peripheral blood mononuclear cells (PBMC) isolated from healthy and repeat breeder cows. The mRNA expression of IFNT-response makers (*ISG15* and *MX2)* were significantly increased by IFNT treatment in a dose-dependent manner in both groups. Although treatment with IFNT promoted the expression of *IDO* in PBMCs from both groups, it did so at a substantially reduced rate among the repeat breeder cows, suggesting that decreased levels of kynurenine may relate to the reduced IDO expression in repeat breeder cows.

**Conclusions:**

These findings provide valuable information towards the identification of critical biomarkers for repeat breeding syndrome in cattle.

**Supplementary Information:**

The online version contains supplementary material available at 10.1186/s12917-021-02755-7.

## Background

Repeat breeder cows are defined as sub-fertile animals without anatomical or infectious abnormalities that do not become pregnant after three or more breeding attempts [1]. The fertility of lactating dairy cows has been declining gradually worldwide. Repeat breeding is considered to be one of the economically important reproductive disorders in cattle, as it results in an increased number of inseminations and associated costs, and an increase in the number of days open. The physiological cause of the repeat breeding syndrome is complex and multifactorial; repeat breeder cattle have multiple dysfunctions within the endocrine system, including abnormal levels of progesterone, estradiol, epithelial growth factor, and luteinizing hormone, but hormonal treatments in repeat breeder cow cannot completely improve reproductive function [2–4]. Although the ability to identify repeat breeder cattle is one of the keys to efficient early therapy and farm management, repeat breeding syndrome remains largely unmanageable due to the lack of informative specific factors.

Metabolomics involves the quantitative measurement of a global set of low molecular weight metabolites in a biological fluid [5]. Metabolomics data represent a downstream of outcome of systems biology that has drawn significant interest for its capacity to unravel essential biological processes [6]. As such, findings from metabolomics studies may help to identify potential biomarkers of given phenotypes related to health and diseases, including pregnancy and pregnancy-associated dysfunctions [7]. Within cattle populations, metabolomics analysis has been utilized to provide assessments of the quality of embryos and oocytes to identify interactions between mother and embryo, and to determine differences in fertility and infertility using plasma, uterine luminal fluid, and seminal plasma [5, 6, 8, 9]. However, currently, there are no published studies that have reported metabolomics-based differences between healthy and repeat breeder cows.

In ruminants, embryonic trophoblast cells produce and secrete interferon tau (IFNT) which is a well-known pregnancy recognition signal [10]. IFNT suppresses luteolysis via inhibition of release of prostaglandin (PG) F_2α_ from the uterus, resulting in the establishment of pregnancy [11, 12]. In addition to its intrauterine function, IFNT passes through the endometrium and enters the uterine vein [13], where it plays a crucial role in the transformation of tissues during pregnancy via its actions on various components of the immune system [14]. Kimura et al. [15] showed that after transfer of an elongating embryo on day 14, pregnancy rates of the repeat breeder cows were dramatically lower than among their healthy counterparts. Moreover, endometrial gene expression profiles can also distinguish between repeat breeders and otherwise healthy cows [16]. Therefore, we hypothesized that the uterine environment of repeat breeders differ significantly from that of healthy cattle and are likely to be less suitable for supporting successful embryo development.

To the best of our knowledge, there are very few studies that have explored metabolomic profiling in repeat breeder cattle. In the present study, we conducted comprehensive metabolomics profiling of both blood plasma and uterine luminal fluids from healthy and repeat breeder cows using capillary electorophoresis-time of flight / mass spectrometry (CE-TOFMS) and liquid chromatography (LC)-TOFMS. We also examined responses to IFNT among distinct populations of immune cells isolated from healthy and repeat breeder cows.

## Methods

### Animal selection for metabolomics analysis

All the experimental procedures complied with the Guidelines for the Care and Use of Agricultural Animals of Tokyo University of Agriculture, and all the animal protocols were approved by the institutional animal experiment committee (30098).

For metabolomic analysis, Holstein cows housed at the farm of Nippon Veterinary and Life Science University and Kanagawa Prefectural Livestock Industry Technology Center were used. In the present study, healthy cows (*n* = 5, parity 1–8, 4–10 years old, average 650 kg) and repeat breeder cows (n = 5, parity 1–4, 4–8 years old, average 660 kg) were used. Body condition scores of these cows were similar between both groups (the range of 2.75–3.25) and during experiment, no antibiotics were used for these cows after parturition.

Healthy cows were selected and defined by following: After normal parturition, estrous synchronization was performed. Briefly, estradiol benzoate (Ovahormon, 2 mg, Aska pharmaceutical, Tokyo, Japan) was injected intramuscularly (i.m.) and a progesterone preparation (controlled internal drug release (CIDR), Zoetis Japan, Tokyo, Japan) was inserted into the vagina. After 9 days, CIDR was removed and PGF_2α_ (Lutalyse, 25 mg i.m., Zoetis Japan) was injected to induce luteolysis and GnRH (Fertirelin, 100 μg i.m., Fujita pharmaceuticals, Tokyo, Japan) was injected to induce ovulation (= day 1) 2 days after PGF_2α_ administration. Then, after confirming ovulation, blood and uterine fluid was collected on day 7 of the estrous cycle from 8 candidate cows. On average, sampling was performed on 64.6 ± 2.5 days (59–74 days) after parturition and mean milk yield per healthy cow was 35.8 ± 1.9 kg (27.5–38.5 kg). After that, these cows were subjected to AI (spontaneous estrous cycle) and individuals that conceived within three attempts at AI were considered to be healthy cows (5 cows were selected as healthy cows). Pregnancy was determined by transrectal palpation on day 40 after insemination.

Repeat breeding Holstein cows were defined by characteristics [1, 17] that include detectable estrous behavior with occasional abnormal estrous cycles, healthy uterus and ovaries as determined by transrectal palpation, and the inability to conceive after four or more AIs following normal estrous behavior. After selection of 5 repeat breeder cows, the estrous cycle was synchronized using the method used for healthy cows; then blood and uterine fluid was collected from these repeat breeder cows on day 7 of the estrous cycle. In our previous investigations, we transferred in vitro fertilized embryo or parthenogenetic embryo on day 7 after estrus following artificial insemination in repeat breeder cows to improve pregnancy recognition [17, 18]. Therefore, in the present study, we collected samples to investigate the condition of blood and uterine fluid in the same timing with previous study. On average sampling was conducted at 317.6 ± 100.2 days (209–479 days) after parturition and mean milk yield per repeat breeder cow was 32.0 ± 1.6 kg (26.8–35.3 kg).

Although the sampling days after parturition were different between both groups, both the healthy and repeat breeder cows were on a similar ration, thereby minimising the effects due to any differences in nutrition. After collecting the sample, the individual was returned to normal breeding.

Prior to sampling of blood and uterine fluid, endometrial cells were collected using sight brush (metric brush, Fujihira Industry Co., LTD., Tokyo, Japan), applied to a microscopic slide and subjected to Giemsa staining. The percentage of polymorphonuclear leukocytes in all samples were less than 5.0%, indicating no subclinical endometritis in both healthy and repeat breeder cows. For the present study, we made certain that the healthy and repeat breeder cows were clinically normal, specifically with no subclinical endometritis detected by cytology and no evidence of clinical ketosis. In addition, none of the cows in this study had a history of hypocalcemia, metritis, retained placenta, or displaced abomasum.

### Blood collection and processing

Blood sample (10 ml, Vacuum blood collection tube containing sodium heparin, Terumo Corporation, Tokyo, Japan) was collected from the jugular vein of each cow. The samples were centrifuged at 3000×g for 20 min at 4 °C. Plasma (500 μl) was collected in 1.5 ml tubes and stored at − 80 °C until further processing for metabolomic analysis.

### Collection of uterine luminal fluid

To collect uterine luminal fluid, the uterine horns ipsilateral to the ovary with corpus luteum (assuming the attachment site) were flushed individually using the technique of embryo recovery on day 7 of the estrous cycle using a balloon catheter (multi eyes type, 16 Fr, 2.0 mm inner core, Fujihira Industry). With reference to a previously reported method [19], each uterine horn was infused with 50 ml PBS (pH 7.4) and fluid was collected. The collected flushing fluid was centrifuged at 1000 rpm for 10 min and the supernatant was stored at -80 °C until further processing.

### Metabolite extraction

Metabolite extraction and metabolome analysis of plasma (4 healthy cows and 5 repeat breeder cows due to sample condition) and uterine fluids (5 healthy cows and 4 repeat breeder cows due to sample condition) were conducted at Human Metabolome Technologies (HMT), Japan. For CE-TOF/MS analysis, 50 μl of plasma were added to 450 μl of methanol and 80 μl of uterine luminal fluid were added to 20 μl of solution including internal standards. The solution was mixed thoroughly with 500 μl chloroform and 200 ml Milli-Q water and centrifuged at 2300×g for 5 min at 4 °C. The upper aqueous layer was filtered through Millipore 5 kDa molecular weight cut-off membrane at 9100×g for 120 min at 4 °C to remove macromolecules. The filtrate was then centrifuged, and reconstituted in 25 μl Milli-Q water prior to CE-TOF/MS analysis.

For LC-TOF/MS analysis, 100 μl plasma or 200 μl uterine luminal fluid samples were added to 300 μl or 600 μl acetonitrile with 1% formic acid, respectively, each containing an internal standard solution. The solution was then mixed and centrifuged at 2300×g for 5 min at 4 °C. The supernatant was applied to a Hybrid SPE phospholipid cartridge; the filtrate was dried with nitrogen gas and reconstituted in 200 μl of 50% isopropanol prior to LC-TOF/MS.

### Metabolome analysis

Metabolome analysis was conducted by CE-TOF/MS and LC-TOF/MS using Advanced Scan Plus (HMT) based methods described previously. Data obtained from both CE-TOF/MS and LC-TOF/MS studies were processed by MasterHands (Keio University, Tsuruoka, Yamagata, Japan); information extracted included *m/z*, peak area, and migration time (MT) for CE-TOF/MS, and retention time (RT) for LC-TOF/MS. Signal peaks corresponding to isotopomers, adduct ions, and other product ions of known metabolites were excluded from further analysis. The remaining peaks were annotated according to the HMT metabolite database based on their *m/z* values together with their respective MTs or RTs. The areas of the annotated peaks were then normalized to the levels of the internal standard levels and sample volumes for relative quantification. The relative area value of the detected peak was calculated, and the detection limit was about 1 μM.

### For in vitro experiment: preparation of blood immune cells and treatment of IFNT

Blood samples were collected to separate peripheral blood mononuclear cells (PBMC) from healthy (*n* = 4) and repeat breeder (*n* = 3) cows. PBMCs were isolated using Lymphoprep (Axis-Shield, Oslo, Norway) as described in a previous study [20]. Isolated PBMCs were resuspended in RPMI 1640 medium (Life Technologies Corporation, Carlsbad, CA, USA) with 5% fetal bovine serum (FBS; HyClone, GE Healthcare UK Ltd., Buckinghamshire, England). Next, PBMCs were treated with recombinant bovine IFNT (0.1, 1, 10, or 100 ng/ml) [21], for 6 h at 38 °C. After incubation, cells were collected using ISOGEN II (Nippon Gene Co., Ltd., Tokyo, Japan) to analyze mRNA expression, and stored at -80 °C until analysis.

### RNA extraction, cDNA production, and real-time PCR

Total RNA was prepared using ISOGEN II according to the manufacturer’s instructions. cDNA production were performed with a commercial kit (ReverTra Ace; Toyobo Co., Ltd., Osaka, Japan). Real-time quantitative PCR was performed with the CFX Connect™ Real Time PCR system (Bio-Rad, Hercules, CA, USA) and a commercial kit (Thunderbird SYBR qPCR Mix; Toyobo Co., Ltd.) to detect the mRNA expressions of *Interferon stimulated gene (ISG)15*, *MX dynamin like GTPase 2* (*MX2*), *indoleamine 2,3-dioxygenase* (*IDO*), or *glyceraldehyde 3-phosphate dehydrogenase* (*GAPDH*). The primers used for real-time PCR were as follows: forward, 5′-GGTATGATGCGAGCTGAAGCACTT-3′ and reverse, 5′-ACCTCCCTGCTGTCAAGGT-3′ for ISG15 (accession no. NM_174366); forward, 5′- GGACAGCGGAATCATCAC-3′ and reverse, 5′- CTCCCGCTTTGTCAGTTTCAG-3′ for MX2 (accession no. NM_173941); forward, 5′- CGAATATACTTGTCTGGTTGG − 3′ and reverse, 5′- GGAGAACATCAAAGCACTG − 3′ for IDO (accession no. NM_00101866); forward, 5′- ACAGTCAAGGCAGAGAACGG-3′ and reverse, 5′- CCACATACTCAGCACCAGCA-3′ for GAPDH (accession no. NM_001034034). RT-qPCR was performed in duplicate with a final reaction volume of 20 μl containing 10 μl of SYBR Green, 7.8 μl of distilled water, 0.1 μl of 100 μM forward and reverse primers, and 2 μl of cDNA template. The amplification program consisted of a 5 min denaturation at 95 °C followed by 40 cycles of amplification (95 °C for 10 s, 56-60 °C for 10 s, and 72 °C for 20 s). The expression levels of each target gene were normalized to the corresponding GAPDH threshold cycle (CT) values using the ΔΔ CT comparative method [22]. The relative amount of each PCR product was also calculated in comparison, using GAPDH as the international standard. To select appropriate house-keeping gene, we checked 4 candidate genes such as GAPDH, β-actin, succinate dehydrogenase complex and Tyrosine 3-Monooxygenase/Tryptophan 5-Monooxygenase Activation Protein Zeta, and we selected GAPDH because of stable expression levels without change due to IFNT treatment.

### Statistical analysis

All data are presented as means ± SEM. Metabolome data of plasma and uterine luminal fluid samples were z-value transformed and principal component analysis (PCA) applied using PeakStat (HMT) and the relative area value of the detected peak was calculated. The statistical significance of differences was assessed by Student’s *t*-test or one-way ANOVA followed by Bonferroni’s multiple comparison test. Probabilities less than 5% (*P* < 0.05) were considered significant.

## Results

### Differentially expressed metabolites identified in blood plasma samples from healthy and repeat breeder cows

We analyzed the metabolomics profiles of plasma obtained from 4 healthy cows and 5 repeat breeder cows using both CE-TOF/MS and LC-TOF/MS, and identified 318 differentially expressed metabolites based on their m/z values, MTs and RTs. PCA analysis of plasma metabolites could not clearly separate healthy and repeat breeder cows. Univariate t-test analysis revealed statistically significant differentials for 17 of these metabolites (Table 1). After filtering for metabolites with at least a 2-fold differences, our final list of 8 lesser-detected metabolites included litocholic acid (LA), gycochenodeoxycholic acid (GCDCA), glycolithocholic acid (GLA), taurodeoxycholic acid (TDCA), deoxycholic acid (DCA), taurochenodeoxycholic acid (TCDCA), chenodeoxycholic acid (CDCA), and glycodeoxycholic acid (GDCA) in repeat breeder cows. These data suggest that the level of plasma bile acids in repeat breeder cows are significantly lower than in healthy cows.

**Table 1:** Blood metabolites found at significantly different levels in the repeat breeder cows (RBC) when compared to healthy cows (control)

Compound name	Healthy	RBC	Ratio	*P*-value
	Mean	Mean	RBC/Healthy	
Kynurenine	0.002127	0.001630	0.77	0.001
*N*^6^-Methyllysine	0.003122	0.001676	0.54	0.001
2-Hydroxyglutaric acid	0.000102	0.000157	1.53	0.010
α-Tocopherol acetate-2	0.000268	0.000139	0.52	0.017
Lithocholic acid	0.000017	0.000008	0.47	0.019
Arachidonic acid	0.000337	0.000259	0.77	0.020
Glycochenodeoxycholic acid	0.001113	0.000399	0.36	0.021
Glycolithocholic acid	0.000169	0.000060	0.35	0.023
Choline	0.004052	0.006171	1.52	0.023
*cis*-5,8,11,14,17-Eicosapentaenoic acid	0.000062	0.000047	0.75	0.028
Taurodeoxycholic acid	0.004032	0.001855	0.46	0.035
Deoxycholic acid	0.003749	0.001531	0.41	0.036
Taurochenodeoxycholic acid	0.000912	0.000400	0.44	0.038
Homocitrulline	0.000214	0.000130	0.61	0.038
Chenodeoxycholic acid	0.000443	0.000197	0.45	0.039
2-Aminobutyric acid	0.011673	0.007128	0.61	0.043
Glycodeoxycholic acid	0.006179	0.002452	0.40	0.044

Based on these findings, concentrations of additional metabolites that are known to be associated with bile acids were evaluated (Table 2). Cholesterol is a substrate for synthesis of bile acids; however, cholesterol levels were detected at similar levels in plasma samples from healthy and repeat breeder cows. By contrast, in addition to the 8 metabolites noted above (Table 1), 4 additional metabolites including cholic acid (CA), glycocholic acid (GCA), taurocholic acid (TCA), and taurolithocholic acid (TLCA) were found to be lower in plasma samples from repeat breeder cows than that from healthy cows. Importantly, all bile acid metabolites were detected at lower levels in plasma of repeat breeder cows compared to those detected in plasma of healthy cows.

**Table 2:** Blood metabolites of bile acids in the RBC when compared to healthy cows (control)

	Compound name	Healthy	RBC	Ratio	*P*-value
		Mean	Mean	RBC/Healthy	
Bile acid substrate	Cholesterol	0.08541	0.08254	0.97	0.360
Primary bile acids	Cholic acid	0.01172	0.00689	0.59	0.164
	Chenodeoxycholic acid	0.00044	0.00020	0.45	0.039
Conjugated primary bile acids	Glycocholic acid	0.00794	0.00391	0.49	0.058
	Taurocholic acid	0.00330	0.00196	0.59	0.054
	Glycochenodeoxycholic acid	0.00111	0.00040	0.36	0.021
	Taurochenodeoxycholic acid	0.00091	0.00040	0.44	0.038
Secondary bile acids	Deoxycholic acid	0.00375	0.00153	0.41	0.036
	Lithocholic acid	0.00002	0.00001	0.47	0.019
Conjugated secondary bile acids	Glycodeoxycholic acid	0.00618	0.00245	0.40	0.044
	Taurodeoxycholic acid	0.00403	0.00185	0.46	0.035
	Glycolithocholic acid	0.00017	0.00006	0.35	0.023
	Taurolithocholic acid	0.00053	0.00024	0.46	0.106

As shown in Supplementary Table 1, there were no differences in blood metabolites associated with fatty acids including butyric acid, fatty acid metabolism and fat metabolism between healthy and repeat breeder cows.

### Metabolites in uterine luminal fluid of normal and repeat breeder cows

We analyzed the metabolomics profiles of uterine luminal fluids obtained from 5 healthy cows and 4 repeat breeder cows using CE-TOF/MS and LC-TOF/MS. One hundred and sixty-seven metabolites were detected in varying amounts based on their m/z values, MTs and RTs. Unfortunately, none of the differences detected reached statistical significance in uterine luminal fluids between repeat breeder and healthy cows. Therefore, we selected 9 metabolites that tended to fluctuate (*P* < 0.2) as shown in Table 3. In uterine luminal fluids, several bile acid metabolites including GDCA, CA, GCDCA, DCA and GCA, were detected at lower levels in uterine fluids of repeat breeder cows, similar to what was observed for the plasma metabolites.

**Table 3:** Uterine fluid metabolites found at slightly different levels in the RBC when compared to healthy cows (control)

Compound name	Healthy	RBC	Ratio	*P*-value
	Mean	Mean	RBC/Healthy	
Glycodeoxycholic acid	0.000024	0.000007	0.31	0.088
Cholic acid	0.000032	0.000014	0.44	0.137
Ethanolamine phosphate	0.000048	0.000083	1.74	0.142
Cholesterol sulfate	0.000187	0.000111	0.59	0.152
Glycochenodeoxycholic acid	0.000003	0.000002	0.58	0.157
Taurine	0.000403	0.000917	2.28	0.169
Deoxycholic acid	0.000004	0.000002	0.49	0.177
Creatine	0.000578	0.000974	1.69	0.195
Glycocholic acid	0.000040	0.000018	0.46	0.199

### Responses to IFNT among PBMCs isolated from normal and repeat breeder cows

**Fig. 1:**
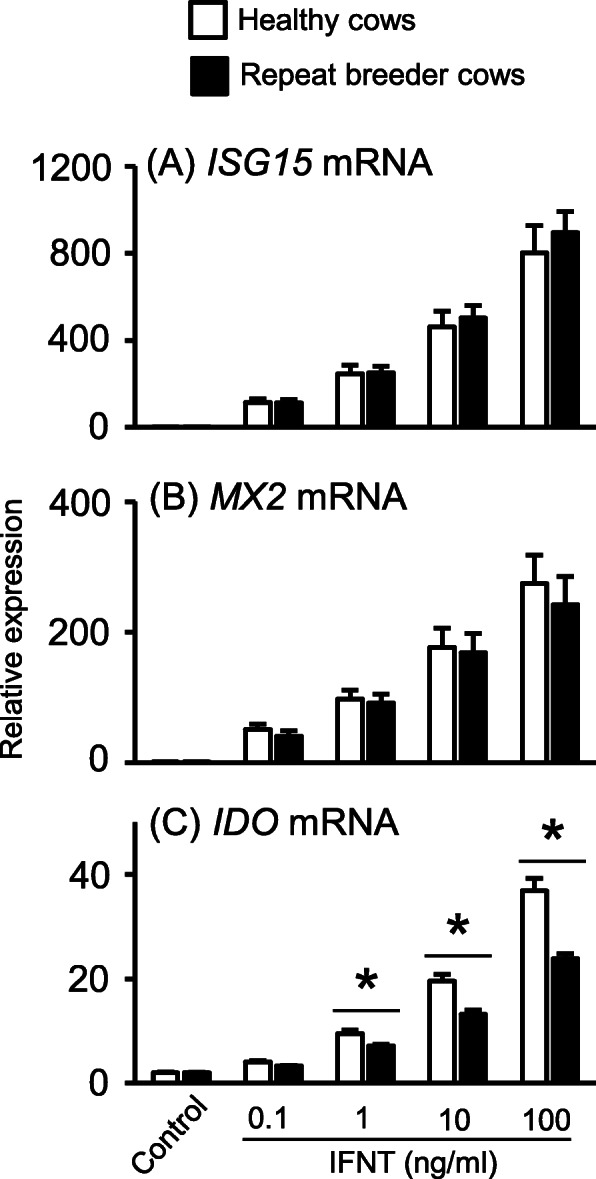
Comparison of IFNT responsivity on PBMC isolated from repeat breeder and healthy cows. **a**-**c** Bovine PBMCs were isolated from repeat breeder (black bar) and healthy (white bar) cows and treated with or without IFNT (0.1–100 ng/ml) for 6 h. Then, ISG15, MX2, and IDO mRNA expression was determined (*n* = 3–4, each group). ‘*’ indicates significant difference (*P* < 0.05) as determined by t test

In Table 1, kynurenine was identified as the metabolite with the most significant variation in the comparison of plasma samples from healthy vs. repeat breeder cows. Kynurenine is a metabolite produced from the essential amino acid tryptophan via the actions of the cytoplasmic enzyme IDO. To establish pregnancy, IDO plays key role in inducing maternal immune tolerance [23] and bovine IFNT can induce IDO activity and mRNA expression in immune cells [24]. Therefore, we isolated PBMCs from healthy and repeat breeder cows and examined their responses to IFNT. The mRNA expression of IFNT-response markers including *ISG15* and *MX2* were significantly increased by treatment with IFNT in a dose-dependent manner, although we observed no differences when comparing responses of healthy and repeat breeder cows (Fig. 1a and b). Interestingly, although IFNT stimulated *IDO* mRNA expression on PBMCs from both healthy and repeat breeder cows, the rate of increase in *IDO* mRNA expression was significantly lower in repeat breeder cows than in healthy cows (Fig. 1c). The basal levels (control) of IDO mRNA expression was same between healthy and repeat breeder cows.

## Discussion

In the present study, we performed metabolomic analysis in an attempt to identify any differences between small molecule metabolites present in biological fluids from healthy and repeat breeder cows; our aim was to identify specific factors diagnostic for repeat breeder syndrome. Although our study did not reveal any potential biomarkers in uterine luminal fluids, we did identify 17 plasma metabolites detected in significantly different levels between healthy and repeat breeder cows. Among them, kynurenine, which was detected in comparatively lower levels in plasma of repeat breeder cows, was identified as the metabolite with the most highly variable concentration. Essentially, similar with the present study, Phillips et al. [5] also found that kynurenine was present in significantly lower levels in plasma of infertile heifers compared to levels detected in samples from fertile heifers. In addition, reduced levels of 2-aminobutyric acid were recognized as candidate biomarkers for poor reproductive performance from the results of the present and previous study [5]. These findings suggest that levels of kynurenine and 2-aminobutyric acid may predict fertility problems including both infertility and repeat breeder syndrome in cattle.

Our study also revealed several interesting and potential biomarker metabolites characteristic of repeat breeder cows, including N6-methyllysine, cis-5,8,11,14,17-eicosapentaenoic acid (EPA), and homocitrulline (L-citrulline). N6-methyllysine is a naturally occurring amino acid found in biofluids; lysine methylation is an essential feature of epigenetic transcriptional regulation, which may be critical to promote normal development and functioning of female reproduction [25]. EPA is an omega-3 long-chain fatty acid that is essential for proper fetal development and healthy aging; supplementation with omega-3 long-chain fatty acids during pregnancy has been linked to decreased immune responses in infants [26]. Homocitrulline is similar in structure to the parent citrulline, and includes one additional methylene group [19]. Interestingly, higher serum citrulline levels have been suggested as potential biomarkers for fertility in bull [27]. Comparatively lower levels of these metabolites may be markers for reproductive pathology in repeat breeder cows, although their role is largely unknown and needs further investigation.

Interestingly, levels of all detectable bile acids in plasma and uterine luminal fluids were lower in repeat breeder cows than in fluids from their healthy counterparts, especially in plasma (Tables 2 and 3). Bile acids are synthesized from cholesterol in the liver and are released post-prandially into the intestines to facilitate the absorption of dietary lipids and lipid-soluble vitamins [28]. Primary bile acids (CA and CDCA) are synthesized in the liver and secondary bile acids (DCA and LCA) are converted by the intestinal flora. Also, bile acids function as conjugates of glycine and taurine. In the present study, no differences in plasma cholesterol levels were detected between repeat breeder and healthy cows; these results suggested two possibilities: (1) bile acid synthesis may be reduced in repeat breeder cows due to some hepatic abnormalities or (2) there may be unrecognized dysbiosis, i.e., aberrant composition of the intestinal flora in repeat breeder cows. With respect to the first possibility, Crociati et al. [29] reported abnormal fatty infiltration in the liver as well as uterine and ovarian tissues in repeat breeder dairy cows, suggesting that dysfunctional lipid metabolism and lipid infiltration in the liver may lead to impaired fertility. Moreover, following embryo transfer in recipient cows, the pregnancy rates of cows with diarrheal feces were significantly lower than those of cows with a normal feces [30], suggesting that dysbiosis of the intestinal flora may be associated with dysregulation of secondary bile acid synthesis and ultimately of reproductive function but these need to be investigated further.

A new role for bile acids is drawing attention that bile acids are also crucial signaling molecule that can regulate and activate cell surface and nuclear receptors. Bile acid-mediated signaling has been linked to improvements in metabolic syndromes and control of inflammation. The two best characterized bile acid receptors are transmembrane G protein-coupled receptor-5 (TGR5) and the nuclear receptor farnesoid X receptor (FXR). For example, bile acid-mediated activation via TGR5 or FXR has a direct impact on physiological pathways that regulate energy expenditure, glucose tolerance, and inflammatory cytokine production [28, 31, 32]. Conversely, other reports suggest that bile acid-mediated signaling serves to promote inflammation and disease development [33]. In support of this latter hypothesis, obesity-associated increases in the level of bile acids, most notably DCA, trigger inflammatory cytokine production, leading to the development of hepatic cancer, sepsis, and colitis in mouse models [34, 35]. Taken together, these findings indicate that bile acids have a variety of context-dependent roles depending on the given circumstances in individual cells and tissues. On the other hand, there is some available information on relationship between bile acids and female reproduction, including reports on bile acids that are present in follicular fluid of cattle and humans [36, 37]. Specifically, the bile acid tauroursodeoxycholic acid promotes embryo quality, resulting in an increased rate of implantation and live birth in mice [38]. However, the detailed roles of bile acids and their actions with respect to reproductive function remain unknown. In the future, we will explore the impact of bile acids on reproductive functions including implantation and immune tolerance; we are specifically interested in understanding the significance of the comparative low levels of bile acids found in plasma samples from repeat breeder cows.

Tryptophan is catabolized primarily via the kynurenine and serotonin pathways. Kynurenine is a metabolite of tryptophan generated by the cytoplasmic enzyme, IDO. Here, although plasma tryptophan levels are comparable, kynurenine levels were lower in repeat breeder cows than in healthy cows; these results suggest that the kynurenine levels detected in this study may reflect differences in expression or activity of IDO. In general, IDO is a key immunomodulatory enzyme that promotes immune tolerance via inhibition of T-cell activation, induction of regulatory T-cells, and suppression of T-cell proliferation through tryptophan catabolism [39]. During pregnancy, there were high levels of IDO expression both in serum and placenta, and inhibition of IDO could result in rejection of allogenic fetuses, indicating that IDO at the maternal-fetal interface is necessary to support pregnancy-related immune tolerance [40, 41]. Interestingly, we showed that the IFNT-mediated increased expression of IDO mRNA was significantly lower in PBMCs isolated from repeat breeder cows compared to those from healthy cows. These data suggested that reduced IDO responses to IFNT may be important factors involved in difficult pregnancies and may lead to the development of repeat breeding in cattle. Actually, low levels of IDO in placenta and immune cells are strongly associated with the occurrence of spontaneous recurrent miscarriages in humans [42, 43].

Recently, investigations based on metabolome analysis of uterine luminal fluid are increasing in numbers. Tribulo et al. [8] reported that most of metabolites in uterine luminal fluid reached peak levels during the embryo transition from morula to the blastocyst stage; these results suggested a role of uterine metabolites in regulating embryonic development in cows. In addition, pregnancy has been associated with remarkable shifts in uterine metabolites in both cows and ewes [9, 44]. Unfortunately, we were unable to identify metabolites that varied significantly in the uterine luminal fluids from repeat breeder and healthy cows in the present study. Further investigations may include some reconsideration of the methods used to collect (volume for flushing) and to concentrate uterine luminal fluids. In addition, in the present study, we studied individual cows that were already identified as repeat breeders, thus, the dates at which postpartum samples were collected for each individual cow varied greatly between repeat breeder and healthy cows. Therefore, we cannot completely rule out the impact of uterine and hepatic recovery conditions after parturition. In our next study, we plan to study the metabolomic profiles of plasma and uterine luminal fluid samples from all cows for a defined period of time post-parturition, so that a metabolomic analysis can be performed separately for healthy and repeat breeder cows classified by date of last conception and according to the number of AI attempts. We consider that this type of controlled analysis will provide additional information on the factors leading to repeat breeder syndrome together with the development of predictive markers.

One limitation of the present study is the small number of animals utilized. In order to improve the accuracy of metabolome analysis, it is necessary to increase the number of individuals. Further, major problems occur in identifying repeat breeder cows by metablome analysis in terms of simplicity and cost-effectiveness. Therefore, future validation using diagnostics such as ELISA kit in a larger sample of animals is required to confirm the usefulness of candidate factors for repeat breeding syndrome in cattle. In addition, we analyzed individuals confirmed as repeat breeder cows, so the sampling time was different from that of healthy cows. In future research, it is important to collect sample before repeat breeder is confirmed and to compare and analyze with individuals confirmed as repeat breeder cows by performing multiple AIs thereafter.

## Conclusion

We utilized metabolomics methodology for quantitative evaluation of metabolites in the blood plasma and uterine luminal fluids of repeat breeder and healthy cows. We identified 17 metabolites in blood plasma that were detected at different levels between repeat breeder and healthy cows; no difference in metabolite levels were detected in uterine luminal fluids. Especially, plasma samples from repeat breeder cows had comparatively lower levels of bile acids and decreased kynurenine levels that may be due to reduced IDO activity. While the number of animals utilized in the present study were limited, we plan to validate and to extend the results from this study in the very near future.

## Supplementary Information

**Additional file 2: Supplementary Table 1.** Blood metabolites associated with fatty acid in the RBC when compared to healthy cows.

## Data Availability

The datasets generated and/or analyzed during the current study are available from the corresponding author on reasonable request.

## Abbreviations

CE-TOFMS: Capillary electorophoresis-time of flight / mass spectrometry
CA: Cholic acid
CDCA: Chenodeoxycholic acid
EPA: Cis-5,8,11,14,17-eicosapentaenoic acid
CIDR: Controlled internal drug release
DCA: Deoxycholic acid
FBS: Fetal bovine serum
GAPDH: Glyceraldehyde 3-phosphate dehydrogenase
GCA: Glycocholic acid
GCDCA: Gycochenodeoxycholic acid
GDCA: Glycodeoxycholic acid
GLA: Glycolithocholic acid
IDO: Indoleamine 2,3-dioxygenase
ISG: Interferon stimulated gene
IFNT: Interferon tau
LC-TOFMS: Liquid chromatography-TOFMS
LA: Litocholic acid
FXR: Nuclear receptor farnesoid X receptor
MX2: MX domain like GTPase 2
PBMC: Peripheral blood mononuclear cells
PG: Prostaglandin
TCA: Taurocholic acid
TCDCA: Taurochenodeoxycholic acid
TDCA: Taurodeoxycholic acid
TLCA: Taurolithocholic acid
TGR5: Transmembrane G protein-coupled receptor-5

## References

[1] Dochi O, Takahashi K, Hirai T, Hayakawa H, Tanisawa M, Yamamoto Y, Koyama H (2008). The use of embryo transfer to produce pregnancies in repeat-breeding dairy cattle. Theriogenology.

[2] Bage R, Gustafsson H, Larsson B, Forsberg M, Rodriguez-Martinez H (2002). Repeat breeding in dairy heifers: follicular dynamics and estrous cycle characteristics in relation to sexual hormone patterns. Theriogenology.

[3] Canu S, Boland M, Lloyd GM, Newman M, Christie MF, May PJ, Christley RM, Smith RF, Dobson H (2010). Predisposition to repeat breeding in UK cattle and success of artificial insemination alone or in combination with embryo transfer. Vet Rec.

[4] Katagiri S, Moriyoshi M (2013). Alteration of the endometrial EGF profile as a potential mechanism connecting the alterations in the ovarian steroid hormone profile to embryonic loss in repeat breeders and high-producing cows. J Reprod Dev.

[5] Phillips KM, Read CC, Kriese-Anderson LA, Rodning SP, Brandebourg TD, Biase FH, Marks ML, Elmore JB, Stanford MK, Dyce PW (2018). Plasma metabolomic profiles differ at the time of artificial insemination based on pregnancy outcome, in Bos taurus beef heifers. Sci Rep.

[6] Velho ALC, Menezes E, Dinh T, Kaya A, Topper E, Moura AA, Memili E (2018). Metabolomic markers of fertility in bull seminal plasma. PLoS One.

[7] Handelman SK, Romero R, Tarca AL, Pacora P, Ingram B, Maymon E, Chaiworapongsa T, Hassan SS, Erez O (2019). The plasma metabolome of women in early pregnancy differs from that of non-pregnant women. PLoS One.

[8] Tribulo P, Balzano-Nogueira L, Conesa A, Siqueira LG, Hansen PJ (2019). Changes in the uterine metabolome of the cow during the first 7 days after estrus. Mol Reprod Dev.

[9] Moraes JGN, Behura SK, Bishop JV, Hansen TR, Geary TW, Spencer TE (2020). Analysis of the uterine lumen in fertility-classified heifers: II. Proteins and metabolitesdagger. Biol Reprod.

[10] Imakawa K, Anthony RV, Kazemi M, Marotti KR, Polites HG, Roberts RM (1987). Interferon-like sequence of ovine trophoblast protein secreted by embryonic trophectoderm. Nature.

[11] Meyer MD, Hansen PJ, Thatcher WW, Drost M, Badinga L, Roberts RM, Li J, Ott TL, Bazer FW (1995). Extension of corpus luteum lifespan and reduction of uterine secretion of prostaglandin F2 alpha of cows in response to recombinant interferon-tau. J Dairy Sci.

[12] Spencer TE, Burghardt RC, Johnson GA, Bazer FW (2004). Conceptus signals for establishment and maintenance of pregnancy. Anim Reprod Sci.

[13] Oliveira JF, Henkes LE, Ashley RL, Purcell SH, Smirnova NP, Veeramachaneni DN, Anthony RV, Hansen TR (2008). Expression of interferon (IFN)-stimulated genes in extrauterine tissues during early pregnancy in sheep is the consequence of endocrine IFN-tau release from the uterine vein. Endocrinology.

[14] Bott RC, Ashley RL, Henkes LE, Antoniazzi AQ, Bruemmer JE, Niswender GD, Bazer FW, Spencer TE, Smirnova NP, Anthony RV, Hansen TR (2010). Uterine vein infusion of interferon tau (IFNT) extends luteal life span in ewes. Biol Reprod.

[15] Kimura K, Matsuyama S, Kojima T (2010). Effect of transfer of cattle elongating embryo to a repeat breeder cow on pregnancy rate and incidence of a return to estrus. Reprod Fertil Dev.

[16] Hayashi KG, Hosoe M, Kizaki K, Fujii S, Kanahara H, Takahashi T, Sakumoto R (2017). Differential gene expression profiling of endometrium during the mid-luteal phase of the estrous cycle between a repeat breeder (RB) and non-RB cows. Reprod Biol Endocrinol.

[17] Funeshima N, Noguchi T, Onizawa Y, Yaginuma H, Miyamura M, Tsuchiya H, Iwata H, Kuwayama T, Hamano S, Shirasuna K (2019). The transfer of parthenogenetic embryos following artificial insemination in cows can enhance pregnancy recognition via the secretion of interferon tau. J Reprod Dev.

[18] Yaginuma H, Funeshima N, Tanikawa N, Miyamura M, Tsuchiya H, Noguchi T, Iwata H, Kuwayama T, Shirasuna K, Hamano S (2019). Improvement of fertility in repeat breeder dairy cattle by embryo transfer following artificial insemination: possibility of interferon tau replenishment effect. J Reprod Dev.

[19] Groebner AE, Rubio-Aliaga I, Schulke K, Reichenbach HD, Daniel H, Wolf E, Meyer HH, Ulbrich SE (2011). Increase of essential amino acids in the bovine uterine lumen during preimplantation development. Reproduction.

[20] Shirasuna K, Matsumoto H, Kobayashi E, Nitta A, Haneda S, Matsui M, Kawashima C, Kida K, Shimizu T, Miyamoto A (2012). Upregulation of interferon-stimulated genes and Interleukin-10 in peripheral blood immune cells during early pregnancy in dairy cows. J Reprod Dev.

[21] Takahashi T, Sakumoto R, Hayashi KG, Hosoe M, Shirai J, Hashizume K (2017). Generation of recombinant bovine interferon tau in the human embryonic kidney cell line and its biological activity. Anim Sci J.

[22] Livak KJ, Schmittgen TD (2001). Analysis of relative gene expression data using real-time quantitative PCR and the 2(−Delta Delta C(T)) method. Methods.

[23] Zong S, Li C, Luo C, Zhao X, Liu C, Wang K, Jia W, Bai M, Yin M, Bao S, Guo J, Kang J (2016). Dysregulated expression of IDO may cause unexplained recurrent spontaneous abortion through suppression of trophoblast cell proliferation and migration. Sci Rep.

[24] Maneglier B, Rogez-Kreuz C, Spreux-Varoquaux O, Malleret B, Therond P, Samah B, Drouet I, Dormont D, Advenier C, Clayette P (2007). Comparative effects of two type I interferons, human IFN-alpha and ovine IFN-tau on indoleamine-2,3-dioxygenase in primary cultures of human macrophages. Fundam Clin Pharmacol.

[25] Chamani IJ, Keefe DL (2019). Epigenetics and female reproductive aging. Front Endocrinol (Lausanne).

[26] Swanson D, Block R, Mousa SA (2012). Omega-3 fatty acids EPA and DHA: health benefits throughout life. Adv Nutr.

[27] Kumar A, Kroetsch T, Blondin P, Anzar M (2015). Fertility-associated metabolites in bull seminal plasma and blood serum: 1H nuclear magnetic resonance analysis. Mol Reprod Dev.

[28] Garcia-Irigoyen O, Moschetta A (2017). A novel protective role for FXR against Inflammasome activation and Endotoxemia. Cell Metab.

[29] Crociati M, Di Giacinto F, Manuali E, Stradaioli G, Sylla L, Monaci M, Maulucci G, De Spirito M (2018). Systemic profiling of ectopic fat deposits in the reproductive tract of dairy cows. Theriogenology.

[30] Sasaki K, Tanaka K, Tanimura H, Asakura R, Fukui Y (2008). Relationship between the fecal property and pregnancy rate following embryo transfer in recipient cows. Fukui Prefect Livestock Indust Rep.

[31] Guo C, Xie S, Chi Z, Zhang J, Liu Y, Zhang L, Zheng M, Zhang X, Xia D, Ke Y, Lu L, Wang D (2016). Bile acids control inflammation and metabolic disorder through inhibition of NLRP3 Inflammasome. Immunity.

[32] Iracheta-Vellve A, Calenda CD, Petrasek J, Ambade A, Kodys K, Adorini L, Szabo G (2018). FXR and TGR5 agonists ameliorate liver injury, Steatosis, and inflammation after binge or prolonged alcohol feeding in mice. Hepatol Commun.

[33] Zhao S, Gong Z, Zhou J, Tian C, Gao Y, Xu C, Chen Y, Cai W, Wu J (2016). Deoxycholic acid triggers NLRP3 Inflammasome activation and aggravates DSS-induced colitis in mice. Front Immunol.

[34] Yoshimoto S, Loo TM, Atarashi K, Kanda H, Sato S, Oyadomari S, Iwakura Y, Oshima K, Morita H, Hattori M, Honda K, Ishikawa Y (2013). Obesity-induced gut microbial metabolite promotes liver cancer through senescence secretome. Nature.

[35] Hao H, Cao L, Jiang C, Che Y, Zhang S, Takahashi S, Wang G, Gonzalez FJ, Farnesoid X Receptor regulation of the NLRP3 Inflammasome underlies cholestasis-associated Sepsis. Cell Metab 2017; 25:856–867 e855. 10.1016/j.cmet.2017.03.007 PMC6624427 28380377

[36] Sanchez-Guijo A, Blaschka C, Hartmann MF, Wrenzycki C, Wudy SA (2016). Profiling of bile acids in bovine follicular fluid by fused-core-LC-MS/MS. J Steroid Biochem Mol Biol.

[37] Nagy RA, van Montfoort AP, Dikkers A, van Echten-Arends J, Homminga I, Land JA, Hoek A, Tietge UJ (2015). Presence of bile acids in human follicular fluid and their relation with embryo development in modified natural cycle IVF. Hum Reprod.

[38] Lin T, Diao YF, Kang JW, Lee JE, Kim DK, Jin DI (2015). Tauroursodeoxycholic acid improves the implantation and live-birth rates of mouse embryos. Reprod Biol.

[39] Munn DH, Mellor AL (2016). IDO in the tumor microenvironment: inflammation, counter-regulation, and tolerance. Trends Immunol.

[40] Yamazaki F, Kuroiwa T, Takikawa O, Kido R (1985). Human indolylamine 2,3-dioxygenase. Its tissue distribution, and characterization of the placental enzyme. Biochem J.

[41] Munn DH, Zhou M, Attwood JT, Bondarev I, Conway SJ, Marshall B, Brown C, Mellor AL (1998). Prevention of allogeneic fetal rejection by tryptophan catabolism. Science.

[42] Wei H, Liu S, Lian R, Huang C, Li Y, Chen L, Zeng Y. Abnormal expression of Indoleamine 2, 3-Dioxygenase in human recurrent miscarriage. Reprod Sci. 2019:1933719119833788. 10.1177/1933719119833788 30832549

[43] Miwa N, Hayakawa S, Miyazaki S, Myojo S, Sasaki Y, Sakai M, Takikawa O, Saito S (2005). IDO expression on decidual and peripheral blood dendritic cells and monocytes/macrophages after treatment with CTLA-4 or interferon-gamma increase in normal pregnancy but decrease in spontaneous abortion. Mol Hum Reprod.

[44] Romero JJ, Liebig BE, Broeckling CD, Prenni JE, Hansen TR (2017). Pregnancy-induced changes in metabolome and proteome in ovine uterine flushings. Biol Reprod.

